# Supplementary material for: CD74 Promotes a Pro-Inflammatory Tumor Microenvironment by Inducing S100A8 and S100A9 Secretion in Pancreatic Cancer
Source: Int J Mol Sci. 2023 Aug 20;24(16):12993. doi: 10.3390/ijms241612993 (PMC10455843; doi:10.3390/ijms241612993)
Supplement: Supplementary file 1 [file ijms-24-12993-s001.zip › ijms-2538025-supplementary.pdf]

Supplementary Materials

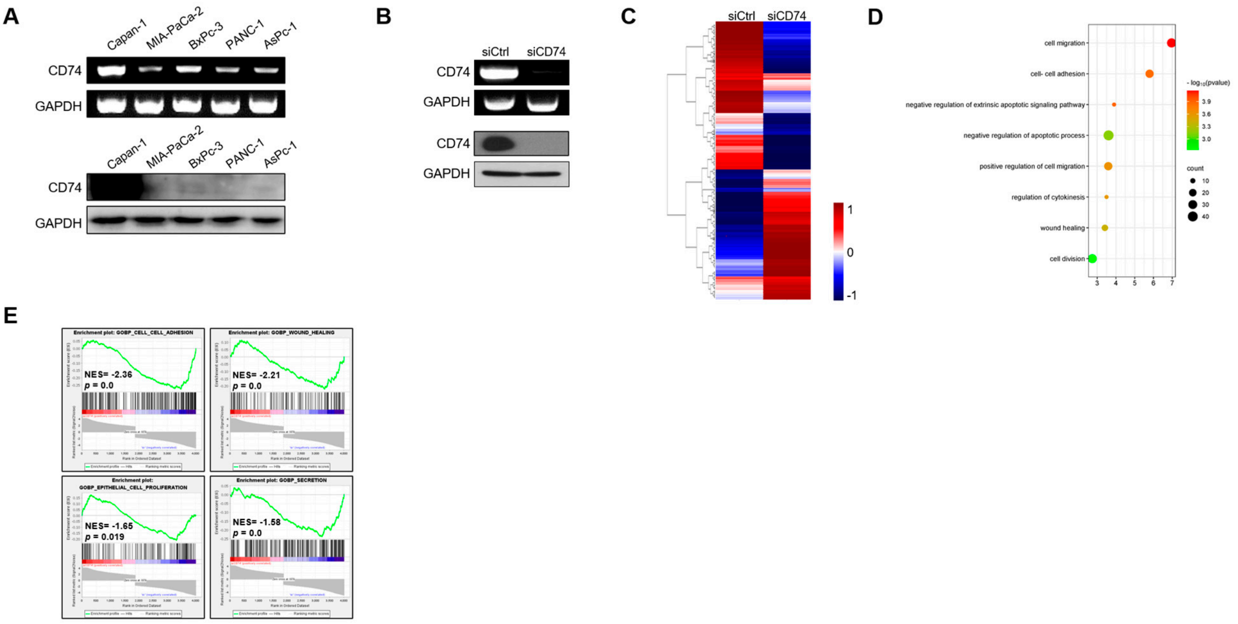

Figure S1. Expression of CD74 across the five PDAC cell lines and RNA-seq results after siRNA-mediated CD74 knockdown. (**A**) The mRNA and protein expression of the five PDAC cell lines after longer exposure time via RT-PCR and western blot. CD74 was expressed across all five cell lines; (**B**) The mRNA and protein expression of CD74 in Capan-1 cells after siRNA-mediated knockdown of CD74; (**C**) Heatmap showing the clustering of gene expression for siControl and siCD74 Capan-1 cells; (**D**) GO analysis of RNA-seq results from the siControl and siCD74 Capan-1 cells. Enriched terms are shown; (**E**) GSEA analyses of RNA-seq results indicating that gene expressions of genes involved in cell adhesion, wound healing, cell proliferation, and secretion were significantly altered from CD74 knockdown.

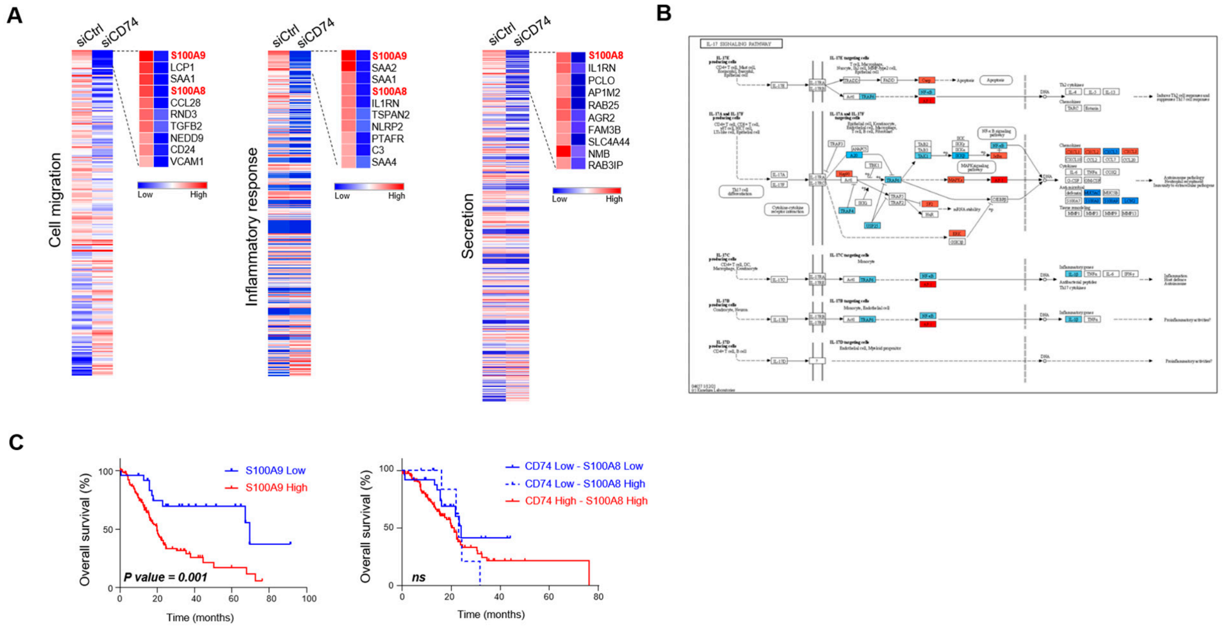

Figure S2. RNA-seq result analysis of CD74 knockdown cells and patient survival analysis. (**A**) Heatmap analysis of RNA-seq results from the siControl and siCD74 Capan-1 cells. The represented genes indicate the genes most significantly reduced by CD74 knockdown in cell migration, inflammatory response, and secretion; (**B**) KEGG pathway analysis of siControl and siCD74 Capan-1 cells indicated significant reduction in the IL-17 signaling pathway in the knockdown cells; (**C**) Kaplan–Meier curves of OS of PDAC patients with high S100A9, low CD74-low S100A9, low CD74-high S100A8, and high CD74-high S100A9 based on TCGA data. ns > 0.05.
